# Case Series on Autosomal Recessive Non-Syndromic Retinitis Pigmentosa Caused by POMGNT1 Mutations with a Report of a New Variant

**DOI:** 10.3390/jcm12247549

**Published:** 2023-12-07

**Authors:** Ami Patel, Ruifeng Cui, James Vernon Odom, Monique Leys

**Affiliations:** Department of Ophthalmology and Visual Sciences, West Virginia University School of Medicine, Morgantown, WV 26506, USA; rucui@hsc.wvu.edu (R.C.); jodom@hsc.wvu.edu (J.V.O.); monique.leys@hsc.wvu.edu (M.L.)

**Keywords:** POMGNT1, non-syndromic retinitis pigmentosa, hypomorphic variant, phenotype variability

## Abstract

Recessive Protein O-linked-mannose beta-1,2-N-acetylglucosaminyltransferase 1 (POMGNT1) mutations can cause early onset muscle–eye–brain disease but have also more recently been associated with non-syndromic Retinitis Pigmentosa. In this case series, we describe three sisters affected by non-syndromic autosomal recessive POMGNT1 retinopathy with a report of a new variant. The three patients received care at West Virginia University Eye Institute, including full ophthalmic examination with additional fundus imaging, optical coherence tomography (OCT), electroretinogram (ERG), and visual field testing. Diagnostic panel testing of 330 genes was also obtained. The proband was seen for cataract evaluation at age 42, and her fundus examination was suggestive of retinitis pigmentosa. Her oldest sister had been treated for acute anterior uveitis with retinal vasculitis. Another sister was diagnosed with multiple sclerosis (MS) and peripheral retinal degeneration. Posterior subcapsular cataracts were diagnosed between age 42 and 55 in all three sisters, each with constricted fields with preserved central vision. We identified one pathogenic POMGNT1 variant (c.751 + 1G > A) and one likely pathogenic variant (c.1010T > C p.Ile337Thr) in all three sisters. A thorough family history and examination of the siblings with genotyping might have led to an earlier diagnosis of retinal inherited disease and avoidance of immunomodulatory treatment in the oldest sibling.

## 1. Introduction

Retinitis pigmentosa (RP) is a group of rare inherited retinal disorders characterized by poor night vision, poor peripheral vision, and an overall decline in visual acuity due to progressive degeneration of rod and cone photoreceptors [1]. RP has been classified as non-syndromic (not affecting other organs or tissues), syndromic (involvement of other neurosensory systems), and systemic (affecting other tissues.) The severity and onset of the disease varies widely and are correlated with different patterns of inheritance. Over 80 genes have been linked to non-syndromic RP [1]. These genes encode proteins that play a vital role in several different processes in the neuroretina and retinal pigment epithelium. Although there is much clinical overlap between RP subtypes, the function of many genes in which pathogenic variants cause RP remains unknown [1,2]. Recessive Protein O-linked-mannose beta-1,2-N-acetylglucosaminyltransferase 1 (POMGNT1) gene mutations have been implicated in muscle–eye–brain disease and, more recently, in the development of non-syndromic RP [3]. POMGNT1 encodes a key enzyme in the O-mannosylation pathway, which has been found to be responsible for assembling and organizing the basal membranes in the neuroretinal system [3,4] and has also been found to be expressed in the photoreceptor inner segment [3,5]. Defects in the O-mannosylation pathway due to hypomorphic mutations of the POMGNT1 gene have been demonstrated to cause RP without extraocular involvement [3]. We extend the limited research on POMGNT1 retinopathy [3] by presenting a case series of three middle-aged sisters with heterozygous POMGNT1 variants. We describe the clinical characteristics and a new POMGNT1 variant for these three sisters with late-onset autosomal recessive RP.

## 2. Materials and Methods

The patients were three sisters who received care at the West Virginia University Eye Institute. The study was approved by the university’s institutional review board (protocol#: 20011172145), and all patients provided written informed consent for their images and test results to be used for publication. Each patient underwent a full ophthalmic examination with additional testing, including fundus imaging, optical coherence tomography (OCT), electroretinogram (ERG), and visual fields. Blood samples were collected from the three sisters and sent to Invitae for diagnostic panel testing (Inherited Retinal Disease Panel of 330 genes), which involves full gene sequencing and deletion/duplication analysis of an extracted genomic DNA sample using next-generation sequencing technology. The sequence analysis covers clinically important regions of each gene, including coding exons and 10 to 20 base pairs of adjacent intronic sequences on either side of the coding exons in the transcripts of interest, depending on the specific gene or test. Four additional family members were examined and genetically tested with the same panel.

## 3. Results

### 3.1. Case 1: 42-Year-Old Female for Cataract Evaluation (Proband)

**Figure 1:**
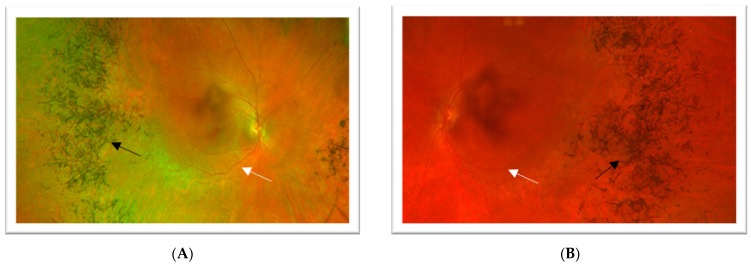
**Case 1**—Ultrawide-field color fundus photograph on Optos California of the right (**A**) and left (**B**) eyes. Both eyes show attenuated arteries (white arrow) and “bone spicules” (black arrow) in the peripheral and mid-peripheral retina. Quality is poor, secondary to posterior subcapsular cataracts. Arrows highlight the pathologic features.

The patient is a 42-year-old female who presented for cataract evaluation in 2022 after experiencing one year of progressive vision blurring, worsening glare with bright lights, and difficulty with night vision. Best corrected visual acuity (BCVA) for the right eye was 20/25-2 (−3.00, +0.75 at 151 degrees), and BCVA for the left eye was 20/50+2 (−2.75, +0.75 at 150 degrees). Anterior segment examination revealed bilateral posterior subcapsular cataracts, with greater central involvement in the left eye. Fundus examination was significant for attenuated arteries and dense retinal hyperpigmented clumping in the peripheral/mid-peripheral retina of both eyes, suggestive of RP (Figure 1).

**Figure 2:**
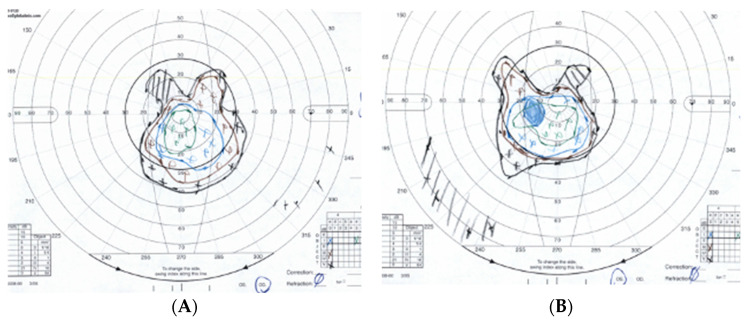
**Case 1**—Goldmann Visual Field of the right (**A**) and left (**B**) eyes. Both visual fields show constricted peripheral fields and preservation of vision in the central 30 degrees.

Subsequently, the patient was referred for Goldmann Visual Field testing (Figure 2).

**Figure 3:**
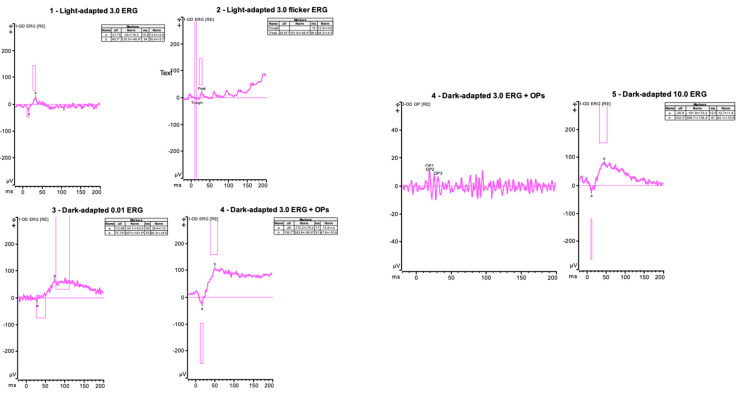
**Case 1**—Full-field electroretinogram of right eye showing results from light-adapted single flash (right eye). Flicker for intensity 3.0 and after 20 min of dark adaptation (right eye for three stimulus intensities). Patient has recordable responses with reduced photopic and scotopic amplitudes. Responses were symmetric in both eyes, but only the results of the right eye are depicted.

A full-field ERG was recorded with DTL electrodes using Diagnosys (Figure 3).

The Invitae inherited retinal disease (IRD) panel of 330 genes test identified one pathogenic variant (POMGNT1 c.751 + 1G > A) and one likely pathogenic variant (POMGNT1 c.1010T > C p.Ile337Thr) in POMGNT1 RP.

### 3.2. Case 2: 45-Year-Old Female with Recurrent Anterior Uveitis and Retinal Vasculitis

**Figure 4:**
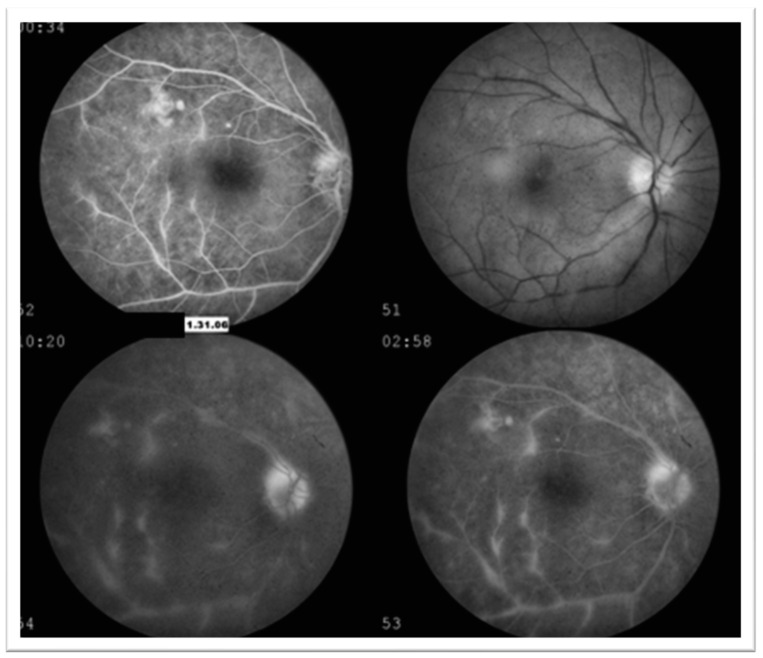
**Case 2**—Fluorescein angiography of the right eye. Hyperfluorescence and leakage along the superior and inferior arcade, indicating inflammation concern for vasculitis.

The second patient is a 45-year-old female who initially presented for an ophthalmologic exam in 2006 for painful red eye. BCVA at that time was 20/30 (−0.25 + 0.50 × 120 = 20/30) for the right eye and 20/20 (−0.25 sph) for the left eye. Clinical examination revealed right eye anterior uveitis. OCT imaging of the macula revealed a center involving macular edema and epiretinal membrane (ERM). Fluorescein angiography (Figure 4) showed leakage along the superior and inferior arcade in the right eye. Bone spicules OD were noted on the dilated exam and thought to be related to vasculitis at that time. Examination findings and imaging of the left eye were within normal limits. The patient was treated with topical steroids and non-steroids (NSAIDs) in the right eye, which resulted in the resolution of symptoms.

Over the next few years, the patient’s acute uveitic flares would be treated with topical steroids and oral NSAIDs. Upon follow-up in 2011, she was noted to have spicular pigmentation and retinal arterial attenuation bilaterally. Intermediate uveitis was noted in the right eye; however, no inflammation was noted in the left eye. These findings, along with the presence of lamellar hole and ERM OD, prompted an electrodiagnostic evaluation with ERG to evaluate for RP. All ERG amplitude parameters were found to be within normal limits, and suspicion of tapetoretinal degeneration remained low. 

**Figure 5:**
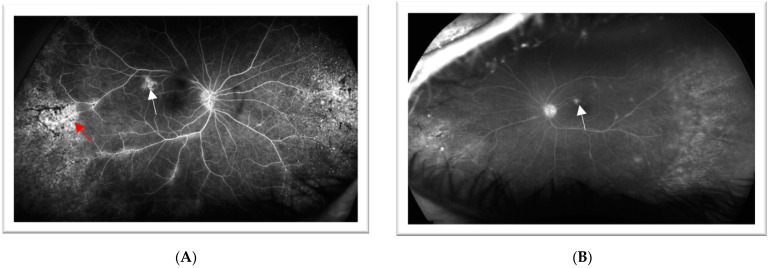
**Case 2**—Fluorescein angiography of the right eye in venous phase (**A**) and left eye in late phase (**B**) showing perivenular leakage (white arrow) and window defects (red arrow) without cystoid macular edema. Arrows highlight the pathologic features.

The patient continued to present intermittently for acute uveitic flare-ups and evaluation of lamellar hole and ERM. She developed a posterior subcapsular cataract of the right eye over the next several years. In 2018, she presented with flashes of light, floaters, and pain in both eyes. Fluorescein angiography at this time showed bilateral perivenular and optic nerve leakage (Figure 5). The patient was started on a high dose of oral prednisone and had a repeat uveitis workup for systemic inflammatory disease. She was HLA-B27 negative. Furthermore, TP-PA, Toxoplasma Bartonella, Lyme, Anca, vasculitis panel, and QuantiFERON were all negative. 

**Figure 6:**
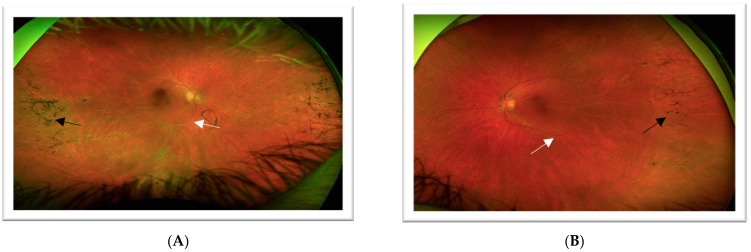
**Case 2**—Ultra-wide field color fundus photograph of the right (**A**) and left (**B**) eyes showing attenuated vessels (white arrow) and hyperpigmented clumping “bone spicules” (black arrow) in the peripheral and mid-peripheral retina. Arrows highlight the pathologic features.

**Figure 7:**
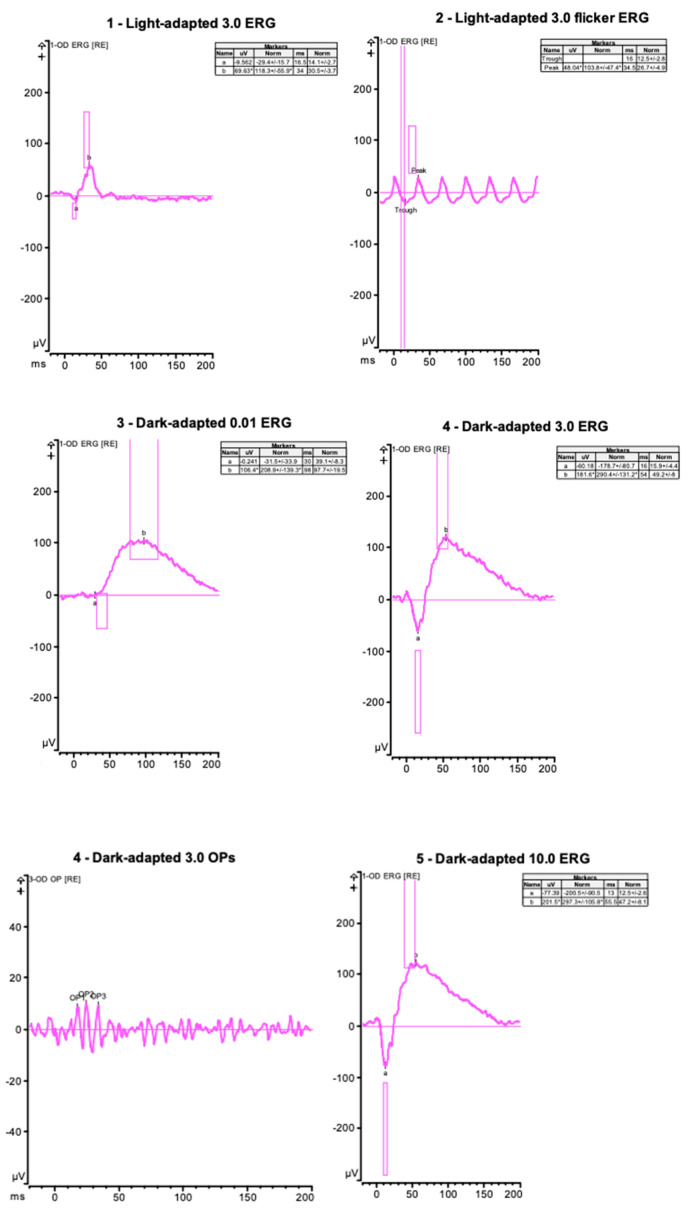
**Case 2**—Full-field electroretinogram of the right eye showing results from light-adapted single flash (right eye). Flicker for intensity 3.0 and after 20 min of dark adaptation (right eye for three stimulus intensities). The dark-adapted a-waves are reduced, and other parameters are within normal limits. Responses were symmetric in both eyes, but only the results of the right eye are depicted.

From 2018 to 2020, the patient was trialed on steroid-sparing immunosuppressive agents (mycophenolate mofetil, azathioprine, and methotrexate) for treatment of her bilateral uveitis, but it was ultimately decided to continue treatment with topical medication only. In 2022, the patient, now aged 61, returned for fundus imaging (Figure 6), repeat ERG (Figure 7), and genetic testing for IRD after her sister was reported to have two variants in POMGNT1 implicated in muscle–eye–brain disease and non-syndromic RP. The 330 gene panel of IRD by Invitae showed the presence of one pathogenic variant (POMGNT1 c.751 + 1G > A) and one likely pathogenic variant (POMGNT1 c.1010T > C p.Ile337Thr).

### 3.3. Case 3: 54-Year-Old Female with Multiple Sclerosis

The final patient is a 52-year-old female who initially presented for ophthalmologic evaluation with neuro-ophthalmology in 2017 after being diagnosed with multiple sclerosis. The anterior segment exam was significant for bilateral posterior subcapsular cataract. A Fundus exam showed bilateral nerve pallor, retinal pigment epithelial changes, and chorioretinal atrophy in both eyes. Humphrey Visual Field testing showed a nonspecific superior depression in the left eye and a general reduction in sensitivity. ERG testing showed reduced responses for all conditions and the presence of artifacts in both eyes. Following cataract surgery, BCVA at her last examination was 20/25 in both eyes. Genetic testing was completed after the patient’s two sisters were found to have POMGNT1-related conditions. Testing was positive for the same gene mutant variants: one pathogenic variant (POMGNT1 c.751 + 1G > A) and one likely pathogenic variant (POMGNT1 c.1010T > C p.Ile337Thr). She also had concurrent MSH6-related Lynch syndrome (HNPCC5) with endometrial adenocarcinoma complicated by stroke and has been unavailable for further follow-up. 

**Figure 8:**
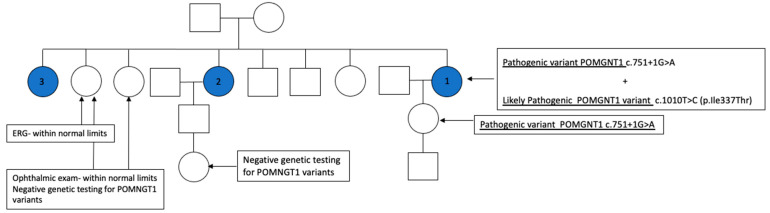
Pedigree chart. Shaded circles represent affected sisters with two POMGNT1 variants and non-syndromic RP. Two additional sisters and the granddaughter of case 2 all tested negative for variants of POMGNT1. The daughter of case 1 was found to have one pathogenic variant (POMGNT1 c.751 + 1G > A) but no clinical findings of RP.

Additional family members were tested, including two unaffected sisters: a sixty-year-old with normal ERG and a forty-six-year-old with normal fundus exam; neither carried a POMGNT1 variant. The daughter of Case 1 did carry the pathogenic variant POMNGT1 c.751 + 1G > A but did not have any abnormalities upon examination. The granddaughter of Case 2 did not carry either POMGNT1 variant. Data from the additional family studies suggest that these variants are likely on opposite chromosomes. Figure 8 shows the pedigree chart for the family.

## 4. Discussion

Over 80 genes have been linked to non-syndromic RP. Recent research by Xu et al. demonstrated that mutations in POMGNT1 can lead to non-syndromic RP [3]. This gene encodes an enzyme that initiates the O-mannosyl glycosylation pathway, a type of protein glycosylation seen in only the muscular and nervous systems [5]. Mutations in POMGNT1 are known causes of muscle–eye–brain disease; however, POMGNT1 has only recently been shown to be associated with non-syndromic RP [6,7]. In this case series, we describe three siblings with non-syndromic POMGNT1 RP who share a pathogenic POMGNT1 variant (c.751 + 1G > A), as well as a novel POMGNT1 variant c.1010T > C (p.Ile337Thr) that is likely pathogenic. The sequence change replaces isoleucine for threonine at codon 337 of the POMGNT1 protein (p.Ile337Thr), resulting in a missense change. Advanced modeling of protein sequence and biochemical properties has been performed at Invitae, but further testing is required to predict the impact of the c.1010T > C variant on protein function. Therefore, c.1010T > C variant has been classified as likely pathogenic. It is unknown if POMGNT1 variant c.1010T > C and variant c.751 + 1G > A are independent of one another or linked as c.751 + 1G > A has not been documented in the literature. However, variant c.751 + 1G > A has been documented to be submitted twice in the ClinVar Database, with the first submission being in May 2019, three years prior to the genetic testing of our proband [8]. 

All three patients became symptomatic in their 5–6th decade, which indicated that they carried hypomorphic variants. The youngest patient (proband) had the most advanced RP, which suggests familial variability and phenotypic heterogeneity of the POMGNT1 genes. Each of our patients presented to different clinics (comprehensive, uveitis, neuro-ophthalmology). The proband initially presented for cataract evaluation and was found to have hallmark findings of RP including posterior subcapsular cataracts, attenuated arteries, and peripheral bone spicules. These findings prompted further visual field, ERG, and genetic testing.

Our second and third cases highlighted the importance of considering RP when creating a differential for uveitis masquerade syndromes. Common symptoms of RP, including mottling/hypopigmentation of the retinal pigment epithelium, cystoid macular edema, and cell or debris in the vitreous, can all also be present in different forms of uveitis. On presentation, our second patient had findings concerning for anterior and posterior uveitis, including AC cell/flare, cystoid macular edema, and fluorescein angiography abnormalities. A uveitis workup for systemic disease was ultimately negative. The third case presented after the diagnosis of multiple sclerosis. At that time, her exam findings (hyperpigmentation/retinal pigment epithelial changes and chorioretinal atrophy in both eyes) were initially thought to be secondary to multiple sclerosis-associated uveitis. It was not until genetic testing was completed that RP was included in the differential. The association between multiple sclerosis and uveitis is well documented, and the incidence of uveitis in multiple sclerosis patients ranges from 0.4 to 26.9% [9]. However, common findings of RP are also seen in various forms of uveitis, and these cases reiterate the importance of considering RP when creating a differential for uveitis masquerade syndromes. Pattern ERG may have added additional information pertaining to the functional reorganization at the optic nerve level; however, unfortunately, these were not conducted in these three cases and represent an area for future research.

Although the association between uveitis and RP is unclear, various reports of uveitis in patients with RP have been published. In 2013, Yoshida et al. found 190 of 509 eyes with RP to have one or more cells in the anterior vitreous cavity [10]. Furthermore, increased levels of numerous proinflammatory cytokines and chemokines were found in the aqueous humor and vitreous fluid of RP patients as compared with levels found in control patients. This could suggest that a chronic inflammatory state underlies the pathogenesis of RP, which can result in various manifestations of anterior, intermediate, and posterior uveitis. In 2018, Dutta Majumder et al. published a retrospective case series reviewing patients with RP who developed uveitis during the course of their disease [11]. They found that the most common uveitis (in 32 eyes of 22 patients with RP and Uveitis) was anterior uveitis (56.2%), followed by intermediate (43.8%). In patients with intermediate uveitis, 41.8% showed extensive vascular leakage.

In summary, this series presents the cases of three sisters with autosomal recessive POMGNT1 mutations leading to non-syndromic RP. These cases add to the limited research on RP resulting from POMGNT1 mutation retinopathy. The finding of a novel POMGNT1 variant, c.1010T > C (p.Ile337Thr), further expands the heterogeneity of RP and suggests an error in the O-mannosylation pathway as one of the many possible pathways resulting in retinal degeneration in RP. All three sisters presented with hallmark findings of RP with no extraocular findings indicative of muscle–eye–brain that is typically associated with POMGNT mutations. In addition, two of the sisters presented with masquerading uveitis findings, suggesting that consideration of RP and potential genetic testing may be beneficial when patients present with uveitis symptoms that are refractory to treatment.

## Data Availability

The data presented in this study are available on reasonable request from the corresponding author. The data are not publicly available due to privacy.
